# Memanti­nium chloride 0.1-hydrate

**DOI:** 10.1107/S1600536809031791

**Published:** 2009-08-19

**Authors:** Wei-Jian Lou, Xiu-Rong Hu, Jian-Ming Gu

**Affiliations:** a Department of Pharmacy, Sir Run Run Shaw Hospital of School of Medicine, Sir Run Run Shaw Institute of Clinical Medicine, Zhejiang University, Hangzhou 310016, People’s Republic of China; b Center of Analysis and Measurement, Zhejiang University, Hangzhou 310028, People’s Republic of China

## Abstract

The crystal structure of the title compound, C_12_H_22_N^+^·Cl^−^·0.1H_2_O, consists of (3,5-dimethyl-1-adamantyl)ammonium chloride (memanti­nium chloride) and uncoordinated water mol­ecules. The four six-membered rings of the memanti­nium cation assume typical chair conformations. The Cl^−^ counter-anion links with the memanti­nium cation *via* N—H⋯Cl hydrogen bonding, forming channels where the disordered crystal water molecules are located. The O atom of the water mol­ecule is located on a threefold rotation axis, its two H atoms symmetrically distributed over six sites; the water mol­ecule links with the Cl^−^ anions *via* O—H⋯Cl hydrogen bonding.

## Related literature

For applications of memantine in medicine, see: Parsons *et al.* (1999 ▶); Tariot *et al.* (2004 ▶). For a related structure, see: Zahid *et al.* (2009 ▶). The H atoms of the ncoordinated water mol­ecule were placed at calculated positions, see: Nardelli (1999 ▶).
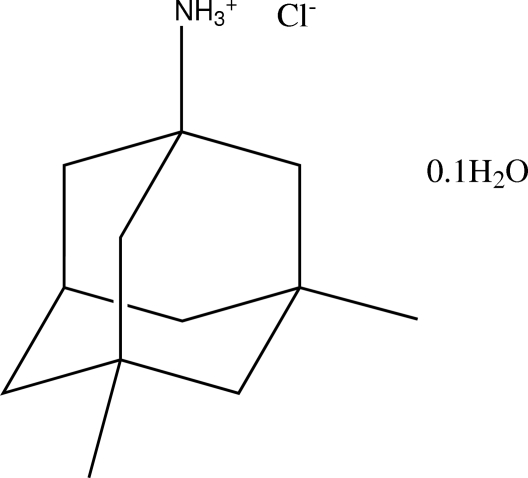


## Experimental

### 

#### Crystal data

C_12_H_22_N^+^·Cl^−^·0.1H_2_O
*M* *_r_* = 217.56
Trigonal, 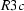

*a* = 28.3787 (11) Å
*c* = 8.5236 (4) Å
*V* = 5944.8 (4) Å^3^
*Z* = 18
Mo *K*α radiation
μ = 0.26 mm^−1^
*T* = 294 K
0.41 × 0.18 × 0.16 mm

#### Data collection

Rigaku R-AXIS RAPID diffractometer
Absorption correction: multi-scan (*ABSCOR*; Higashi, 1995 ▶) *T*_min_ = 0.888, *T*_max_ = 0.959
18491 measured reflections
2845 independent reflections
1671 reflections with *I* > 2σ(*I*)
*R*_int_ = 0.043

#### Refinement

*R*[*F* ^2^ > 2σ(*F* ^2^)] = 0.034
*wR*(*F* ^2^) = 0.104
*S* = 1.09
2845 reflections
132 parameters
1 restraint
H-atom parameters constrained
Δρ_max_ = 0.28 e Å^−3^
Δρ_min_ = −0.31 e Å^−3^
Absolute structure: Flack (1983 ▶), 1329 Friedel pairs
Flack parameter: 0.06 (9)

### 

Data collection: *PROCESS-AUTO* (Rigaku, 2006 ▶); cell refinement: *PROCESS-AUTO*; data reduction: *CrystalStructure* (Rigaku, 2007 ▶); program(s) used to solve structure: *SHELXS97* (Sheldrick, 2008 ▶); program(s) used to refine structure: *SHELXL97* (Sheldrick, 2008 ▶); molecular graphics: *ORTEP-3 for Windows* (Farrugia, 1997 ▶); software used to prepare material for publication: *WinGX* (Farrugia, 1999 ▶).

## Supplementary Material

Crystal structure: contains datablocks global, I. DOI: 10.1107/S1600536809031791/xu2585sup1.cif

Structure factors: contains datablocks I. DOI: 10.1107/S1600536809031791/xu2585Isup2.hkl

Additional supplementary materials:  crystallographic information; 3D view; checkCIF report

## Figures and Tables

**Table 1:** Hydrogen-bond geometry (Å, °)

*D*—H⋯*A*	*D*—H	H⋯*A*	*D*⋯*A*	*D*—H⋯*A*
N1—H1*A*⋯Cl1^i^	0.89	2.26	3.147 (3)	176
N1—H1*B*⋯Cl1^ii^	0.89	2.28	3.161 (2)	171
N1—H1*C*⋯Cl1	0.89	2.26	3.148 (3)	175
O1—H1*E*⋯Cl1^ii^	0.86	2.62	3.486 (17)	179
O1—H1*F*⋯Cl1	0.91	2.93	3.81 (2)	163

Symmetry codes: (i) 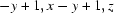; (ii) 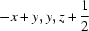.

**Fig. 1.**
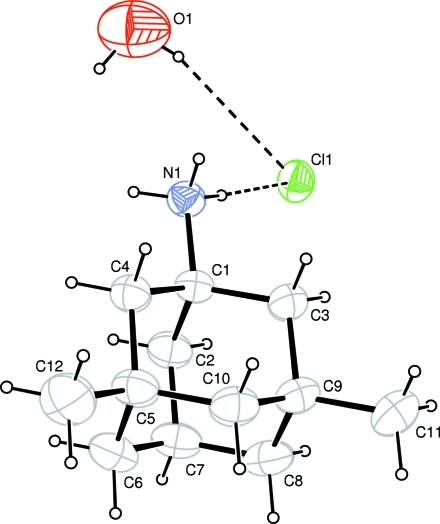
Molecular structure of the title compound showing atom-labelling scheme and displacement ellipsoids at 30% probability level. H atoms are shown as small circles of arbitary radii. Dashed lines indicate the hydrogen bonding.

**Fig. 2.**
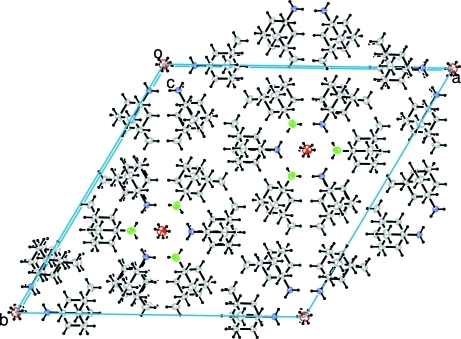
The unit cell packing diagram of the title compound.

## supplementary crystallographic information

### Comment

The title compound is one of a small group of tricycle antiviral drugs (TVA). Memantine also provides good and persistent activation of central nervous *N*-methyl-D-aspartate (NMDA) receptors, and, thus can be used in the treatment of Parkinson's disease and Alzheimer's disease (Parsons *et al.*, 1999; Tariot *et al.*, 2004).

In the asymmetric unit of the crystal structure of the title compound, there are one mamentinium cation, one Cl^-^ anion and 0.10 lattice water molecule. The expected proton transfer from hydrochloric acid to N1 atom of amino group occurs. The four six-membered rings of the memantinium cation assume typical chair conformations, which is comparable with that found in related structures (Zahid *et al.*, 2009). The Cl^-^ counter-anion links with the memantinium cation via N—H···Cl hydrogen bonding (Fig. 1). The lattice water molecules are located on the channels formed by memantininum cations and Cl^-^ anions (Fig. 2). The O atom of lattice water molecule is located at the threefold rotation axis, and its two H atoms are symmetrically distributed over six sites and linked to Cl^-^ anions via O—H···Cl hydrogen bonding (Table 1).

### Experimental

The crude product is supplied by Zhejiang Apeloa Pharmaceutical Co.,LTD. It was recrystallized from ethanol solution, giving colorless crystals of (1) suitable for X-ray diffraction.

### Refinement

Site occupancy factor of the water O1 atom was refined to 0.093 and fixed as 0.1 at the final cycles of refinement. The two H atoms of the water molecule were placed at calculated positions (Nardelli, 1999), and refined as riding in as-found relative positions with U_iso_(H) = 1.5U_eq_(O). Other H atoms were placed in calculated positions with C—H = 0.96–0.98 Å and N—H = 0.89 Å, and included in the refinement in riding model, with U_iso_(H) = 1.2U_eq_(C) and 1.5U_eq_(N).

### Crystal data

**Table:**

C_12_H_22_N·Cl·0.1(H_2_O)	*D*_x_ = 1.094 Mg m^−^^3^
*M**_r_* = 217.56	Mo *K*α radiation, λ = 0.71073 Å
Trigonal, *R*3*c*	Cell parameters from 10816 reflections
Hall symbol: R 3 -2"c	θ = 3.2–27.4°
*a* = 28.3787 (11) Å	µ = 0.26 mm^−^^1^
*c* = 8.5236 (4) Å	*T* = 294 K
*V* = 5944.8 (4) Å^3^	Block, colorless
*Z* = 18	0.41 × 0.18 × 0.16 mm
*F*(000) = 2142	

### Data collection

**Table:**

Rigaku R-AXIS RAPID diffractometer	2845 independent reflections
Radiation source: rolling anode	1671 reflections with *I* > 2σ(*I*)
graphite	*R*_int_ = 0.043
Detector resolution: 10.00 pixels mm^-1^	θ_max_ = 27.4°, θ_min_ = 3.2°
ω scans	*h* = −36→36
Absorption correction: multi-scan (*ABSCOR*; Higashi, 1995)	*k* = −36→36
*T*_min_ = 0.888, *T*_max_ = 0.959	*l* = −11→9
18491 measured reflections	

### Refinement

**Table:**

Refinement on *F*^2^	Hydrogen site location: inferred from neighbouring sites
Least-squares matrix: full	H-atom parameters constrained
*R*[*F*^2^ > 2σ(*F*^2^)] = 0.034	*w* = 1/[σ^2^(*F*_o_^2^) + (0.0331*P*)^2^ + 3.5112*P*] where *P* = (*F*_o_^2^ + 2*F*_c_^2^)/3
*wR*(*F*^2^) = 0.104	(Δ/σ)_max_ = 0.001
*S* = 1.09	Δρ_max_ = 0.28 e Å^−^^3^
2845 reflections	Δρ_min_ = −0.31 e Å^−^^3^
132 parameters	Extinction correction: *SHELXL97* (Sheldrick, 2008), Fc^*^=kFc[1+0.001xFc^2^λ^3^/sin(2θ)]^-1/4^
1 restraint	Extinction coefficient: 0.00185 (17)
Primary atom site location: structure-invariant direct methods	Absolute structure: Flack (1983), 1329 Friedel pairs
Secondary atom site location: difference Fourier map	Flack parameter: 0.06 (9)

### Special details

**Table:**

Geometry. All esds (except the esd in the dihedral angle between two l.s. planes) are estimated using the full covariance matrix. The cell esds are taken into account individually in the estimation of esds in distances, angles and torsion angles; correlations between esds in cell parameters are only used when they are defined by crystal symmetry. An approximate (isotropic) treatment of cell esds is used for estimating esds involving l.s. planes.
Refinement. Refinement of *F*^2^ against ALL reflections. The weighted *R*-factor *wR* and goodness of fit *S* are based on *F*^2^, conventional *R*-factors *R* are based on *F*, with *F* set to zero for negative *F*^2^. The threshold expression of *F*^2^ > σ(*F*^2^) is used only for calculating *R*-factors(gt) *etc*. and is not relevant to the choice of reflections for refinement. *R*-factors based on *F*^2^ are statistically about twice as large as those based on *F*, and *R*- factors based on ALL data will be even larger.

### Fractional atomic coordinates and isotropic or equivalent isotropic displacement parameters (Å^2^)

**Table:**

	*x*	*y*	*z*	*U*_iso_*/*U*_eq_	Occ. (<1)
N1	0.33309 (10)	0.56273 (9)	0.6470 (3)	0.0597 (6)	
H1A	0.3632	0.5911	0.6098	0.090*	
H1B	0.3320	0.5656	0.7508	0.090*	
H1C	0.3041	0.5620	0.6046	0.090*	
C1	0.33298 (13)	0.51132 (11)	0.6066 (3)	0.0552 (7)	
C7	0.28247 (16)	0.41097 (14)	0.6378 (5)	0.0840 (10)	
H7	0.2498	0.3798	0.6813	0.101*	
C5	0.38468 (14)	0.46190 (13)	0.6423 (4)	0.0727 (9)	
C4	0.38361 (11)	0.51418 (10)	0.6791 (3)	0.0621 (7)	
H4A	0.3833	0.5187	0.7918	0.074*	
H4B	0.4160	0.5453	0.6370	0.074*	
C12	0.43554 (15)	0.46469 (17)	0.7130 (5)	0.1084 (13)	
H12A	0.4674	0.4950	0.6689	0.130*	
H12B	0.4357	0.4317	0.6898	0.130*	
H12C	0.4355	0.4691	0.8246	0.130*	
C2	0.28213 (12)	0.46352 (12)	0.6771 (4)	0.0720 (8)	
H2A	0.2498	0.4621	0.6339	0.086*	
H2B	0.2819	0.4678	0.7899	0.086*	
C10	0.38348 (15)	0.45520 (14)	0.4635 (4)	0.0764 (9)	
H10A	0.4159	0.4856	0.4190	0.092*	
H10B	0.3841	0.4222	0.4388	0.092*	
C3	0.33333 (14)	0.50494 (13)	0.4295 (3)	0.0647 (9)	
H3A	0.3653	0.5360	0.3853	0.078*	
H3B	0.3014	0.5037	0.3844	0.078*	
C6	0.33304 (14)	0.41367 (13)	0.7103 (5)	0.0874 (10)	
H6A	0.3331	0.3801	0.6888	0.105*	
H6B	0.3324	0.4177	0.8232	0.105*	
C9	0.33374 (13)	0.45250 (12)	0.3886 (4)	0.0732 (8)	
C11	0.33487 (18)	0.44638 (16)	0.2099 (4)	0.1064 (14)	
H11A	0.3670	0.4769	0.1678	0.128*	
H11B	0.3032	0.4450	0.1644	0.128*	
H11C	0.3351	0.4134	0.1858	0.128*	
C8	0.28330 (16)	0.40466 (14)	0.4610 (5)	0.0876 (11)	
H8A	0.2509	0.4025	0.4157	0.105*	
H8B	0.2829	0.3711	0.4369	0.105*	
Cl1	0.22918 (3)	0.56280 (3)	0.51793 (11)	0.0756 (2)	
O1	0.3333	0.6667	0.800 (4)	0.181 (9)	0.30
H1E	0.3330	0.6410	0.8557	0.272*	0.10
H1F	0.3070	0.6480	0.7275	0.272*	0.10

### Atomic displacement parameters (Å^2^)

**Table:**

	*U*^11^	*U*^22^	*U*^33^	*U*^12^	*U*^13^	*U*^23^
N1	0.0637 (13)	0.0571 (13)	0.0653 (14)	0.0355 (12)	−0.0009 (11)	0.0000 (11)
C1	0.0562 (17)	0.0471 (16)	0.065 (2)	0.0274 (14)	0.0031 (14)	0.0011 (13)
C7	0.077 (2)	0.0513 (19)	0.112 (3)	0.0234 (17)	0.011 (2)	0.0112 (17)
C5	0.074 (2)	0.0604 (19)	0.094 (3)	0.0408 (18)	−0.0034 (17)	0.0013 (17)
C4	0.0613 (16)	0.0568 (16)	0.0704 (18)	0.0312 (13)	−0.0010 (14)	0.0009 (14)
C12	0.110 (3)	0.110 (3)	0.137 (4)	0.079 (3)	−0.029 (3)	−0.011 (2)
C2	0.0639 (18)	0.0590 (17)	0.088 (2)	0.0269 (15)	0.0116 (15)	0.0076 (15)
C10	0.083 (2)	0.066 (2)	0.091 (2)	0.0453 (18)	0.0076 (18)	−0.0068 (18)
C3	0.074 (2)	0.0591 (18)	0.066 (2)	0.0364 (17)	−0.0033 (17)	−0.0064 (14)
C6	0.102 (3)	0.064 (2)	0.101 (3)	0.0448 (19)	0.009 (2)	0.0171 (18)
C9	0.088 (2)	0.0599 (17)	0.077 (2)	0.0406 (17)	−0.0032 (17)	−0.0150 (15)
C11	0.150 (4)	0.094 (3)	0.085 (3)	0.069 (3)	−0.009 (2)	−0.028 (2)
C8	0.086 (3)	0.0538 (19)	0.115 (3)	0.0293 (19)	−0.011 (2)	−0.016 (2)
Cl1	0.0867 (6)	0.0914 (6)	0.0679 (4)	0.0589 (4)	−0.0076 (5)	−0.0064 (5)
O1	0.145 (9)	0.145 (9)	0.25 (3)	0.073 (4)	0.000	0.000

### Geometric parameters (Å, °)

**Table:**

N1—C1	1.497 (3)	C2—H2A	0.9700
N1—H1A	0.8900	C2—H2B	0.9700
N1—H1B	0.8900	C10—C9	1.516 (5)
N1—H1C	0.8900	C10—H10A	0.9700
C1—C3	1.521 (3)	C10—H10B	0.9700
C1—C2	1.525 (4)	C3—C9	1.534 (4)
C1—C4	1.529 (4)	C3—H3A	0.9700
C7—C8	1.519 (5)	C3—H3B	0.9700
C7—C6	1.529 (5)	C6—H6A	0.9700
C7—C2	1.533 (5)	C6—H6B	0.9700
C7—H7	0.9800	C9—C8	1.526 (5)
C5—C12	1.529 (4)	C9—C11	1.535 (5)
C5—C4	1.532 (4)	C11—H11A	0.9600
C5—C10	1.534 (4)	C11—H11B	0.9600
C5—C6	1.533 (5)	C11—H11C	0.9600
C4—H4A	0.9700	C8—H8A	0.9700
C4—H4B	0.9700	C8—H8B	0.9700
C12—H12A	0.9600	O1—H1E	0.8634
C12—H12B	0.9600	O1—H1F	0.9108
C12—H12C	0.9600		
			
C1—N1—H1A	109.5	C7—C2—H2B	110.0
C1—N1—H1B	109.5	H2A—C2—H2B	108.4
H1A—N1—H1B	109.5	C9—C10—C5	112.8 (3)
C1—N1—H1C	109.5	C9—C10—H10A	109.0
H1A—N1—H1C	109.5	C5—C10—H10A	109.0
H1B—N1—H1C	109.5	C9—C10—H10B	109.0
N1—C1—C3	110.3 (3)	C5—C10—H10B	109.0
N1—C1—C2	108.4 (2)	H10A—C10—H10B	107.8
C3—C1—C2	110.2 (2)	C1—C3—C9	110.2 (3)
N1—C1—C4	108.1 (2)	C1—C3—H3A	109.6
C3—C1—C4	110.2 (2)	C9—C3—H3A	109.6
C2—C1—C4	109.5 (2)	C1—C3—H3B	109.6
C8—C7—C6	109.7 (3)	C9—C3—H3B	109.6
C8—C7—C2	109.8 (3)	H3A—C3—H3B	108.1
C6—C7—C2	109.0 (3)	C7—C6—C5	110.2 (3)
C8—C7—H7	109.4	C7—C6—H6A	109.6
C6—C7—H7	109.4	C5—C6—H6A	109.6
C2—C7—H7	109.4	C7—C6—H6B	109.6
C12—C5—C4	110.2 (3)	C5—C6—H6B	109.6
C12—C5—C10	111.1 (3)	H6A—C6—H6B	108.1
C4—C5—C10	108.2 (2)	C10—C9—C8	108.1 (3)
C12—C5—C6	110.7 (3)	C10—C9—C3	108.3 (3)
C4—C5—C6	108.3 (3)	C8—C9—C3	108.2 (3)
C10—C5—C6	108.2 (3)	C10—C9—C11	110.6 (3)
C1—C4—C5	109.8 (2)	C8—C9—C11	111.3 (3)
C1—C4—H4A	109.7	C3—C9—C11	110.2 (3)
C5—C4—H4A	109.7	C9—C11—H11A	109.5
C1—C4—H4B	109.7	C9—C11—H11B	109.5
C5—C4—H4B	109.7	H11A—C11—H11B	109.5
H4A—C4—H4B	108.2	C9—C11—H11C	109.5
C5—C12—H12A	109.5	H11A—C11—H11C	109.5
C5—C12—H12B	109.5	H11B—C11—H11C	109.5
H12A—C12—H12B	109.5	C7—C8—C9	111.0 (3)
C5—C12—H12C	109.5	C7—C8—H8A	109.4
H12A—C12—H12C	109.5	C9—C8—H8A	109.4
H12B—C12—H12C	109.5	C7—C8—H8B	109.4
C1—C2—C7	108.4 (3)	C9—C8—H8B	109.4
C1—C2—H2A	110.0	H8A—C8—H8B	108.0
C7—C2—H2A	110.0	H1E—O1—H1F	102.8
C1—C2—H2B	110.0		
			
N1—C1—C4—C5	179.2 (2)	C8—C7—C6—C5	59.3 (4)
C3—C1—C4—C5	−60.1 (3)	C2—C7—C6—C5	−61.0 (4)
C2—C1—C4—C5	61.2 (3)	C12—C5—C6—C7	−179.5 (3)
C12—C5—C4—C1	179.4 (3)	C4—C5—C6—C7	59.6 (4)
C10—C5—C4—C1	57.8 (3)	C10—C5—C6—C7	−57.5 (4)
C6—C5—C4—C1	−59.4 (3)	C5—C10—C9—C8	−58.5 (3)
N1—C1—C2—C7	−179.1 (3)	C5—C10—C9—C3	58.5 (3)
C3—C1—C2—C7	60.0 (3)	C5—C10—C9—C11	179.4 (3)
C4—C1—C2—C7	−61.4 (3)	C1—C3—C9—C10	−58.1 (3)
C8—C7—C2—C1	−59.2 (4)	C1—C3—C9—C8	58.8 (3)
C6—C7—C2—C1	61.0 (4)	C1—C3—C9—C11	−179.2 (3)
C12—C5—C10—C9	−179.7 (3)	C6—C7—C8—C9	−59.8 (4)
C4—C5—C10—C9	−58.7 (3)	C2—C7—C8—C9	60.0 (4)
C6—C5—C10—C9	58.5 (3)	C10—C9—C8—C7	58.3 (4)
N1—C1—C3—C9	179.5 (3)	C3—C9—C8—C7	−58.8 (4)
C2—C1—C3—C9	−60.8 (3)	C11—C9—C8—C7	180.0 (3)
C4—C1—C3—C9	60.1 (3)		

### Hydrogen-bond geometry (Å, °)

**Table:**

*D*—H···*A*	*D*—H	H···*A*	*D*···*A*	*D*—H···*A*
N1—H1A···Cl1^i^	0.89	2.26	3.147 (3)	176
N1—H1B···Cl1^ii^	0.89	2.28	3.161 (2)	171
N1—H1C···Cl1	0.89	2.26	3.148 (3)	175
O1—H1E···Cl1^ii^	0.86	2.62	3.486 (17)	179
O1—H1F···Cl1	0.91	2.93	3.81 (2)	163

Symmetry codes: (i) −*y*+1, *x*−*y*+1, *z*; (ii) −*x*+*y*, *y*, *z*+1/2.
